# Supplementary material for: Association between Cerebral Infarction Risk and Medication Adherence in Atrial Fibrillation Patients Taking Direct Oral Anticoagulants
Source: Healthcare (Basel). 2021 Oct 1;9(10):1313. doi: 10.3390/healthcare9101313 (PMC8544438; doi:10.3390/healthcare9101313)
Supplement: Supplementary file 1 [file healthcare-09-01313-s001.zip › healthcare-1387814-supplementary.pdf]

Table S1 The period during which data was collected from each hospital.

Hospitals (belonging to the National Hospital Organization)	**Cerebral infarction group**	**Control group**
Yokohama Medical Center	From Sep 2018 to Feb 2020	Sep and Oct 2018, Jan and Feb 2019
Sagamihara Hospital	From Oct 2018 to Feb 2020	Oct and Nov 2018, Jan and Feb 2019
Mito Medical Center	From Sep 2018 to Feb 2020	Sep and Oct 2018, Jan and Feb 2019
Tokyo Medical Center	From Sep 2018 to Feb 2020	Sep and Oct 2018, Jan and Feb 2019
Tochigi Medical Center	From Oct 2018 to Feb 2020	Oct and Nov 2018, Jan and Feb 2019
Utsunomiya Hospital	From Jul 2019 to Feb 2020	Jul and Aug 2019
Shinshu Ueda Medical Center	From Jul 2019 to Feb 2020	Jul and Aug 2019
